# Reprogramming Urine‐Derived Cells using Commercially Available Self‐Replicative RNA and a Single Electroporation

**DOI:** 10.1002/cpsc.124

**Published:** 2020-09-21

**Authors:** Marga J. Bouma, Christiaan H. Arendzen, Christine L. Mummery, Harald Mikkers, Christian Freund

**Affiliations:** ^1^ LUMC hiPSC Hotel Leiden University Medical Center Leiden The Netherlands; ^2^ Department of Anatomy and Embryology Leiden University Medical Center Leiden The Netherlands; ^3^ Department of Cell and Chemical Biology Leiden University Medical Center Leiden The Netherlands

**Keywords:** induced pluripotent stem cell, reprogramming, self‐replicative RNA, urine

## Abstract

We describe a protocol for efficient generation of human‐induced pluripotent stem cells (hiPSCs) from urine‐derived cells (UDCs) obtained from adult donors using self‐replicative RNA containing the reprogramming factors *OCT3/4*, *SOX2*, *KLF4*, *GLIS1*, and *c‐MYC* (ReproRNA‐OKSGM). After electroporation, transfection efficiency is quantified by measuring OCT3/4‐expressing UDCs using flow cytometry and should be ≥0.1%. hiPSC colonies emerge within 3 weeks after transfection and express multiple pluripotency markers. Moreover, the UDC‐derived hiPSCs are able to differentiate into cells of all three germ layers and display normal karyotypes. ReproRNA‐OKSGM is available commercially and only requires a single transfection step so that the protocol is readily accessible, as well as straightforward. In addition to a detailed step‐by‐step description for generating clonal hiPSCs from UDCs using ReproRNA‐OKSGM, we provide guidance for basic pluripotency characterization of the hiPSC lines. © 2020 The Authors.

**Basic Protocol**: Reprogramming of urine‐derived cells using ReproRNA‐OKSGM

**Support Protocol 1**: Determination of the pluripotency status of hiPSCs by flow cytometry

**Support Protocol 2**: Characterization of functional pluripotency of hiPSCs

## INTRODUCTION

Human‐induced pluripotent stem cells (hiPSCs) are widely used as in vitro tools for modeling congenital diseases, studying early human development and toxicology screens, and also hold promise for regenerative medicine (Bellin, Marchetto, Gage, & Mummery, 2012; Singh, Kalsan, Kumar, Saini, & Chandra, 2015). Since the initial reprogramming of human skin fibroblasts from biopsies by Takahashi and Yamanaka (Takahashi et al., 2007) using retroviral vectors to express the reprogramming factors *OCT3/4*, *SOX2*, *KLF4*, and *c‐MYC*, many other cell types have been reprogrammed with a variety of vectors. Non‐integrating reprogramming vectors are preferred as they circumvent risks of remaining‐ or reactivated transgene expression or altered endogenous gene expression, which may limit utility. Reprogramming conditions are ideally highly reproducible and avoid intermediate culture splitting that could yield mixed, non‐clonal hiPSC colonies. This is important because donors could in principle be mosaic.

Urine‐derived cells (UDCs) can be efficiently isolated non‐invasively from urine samples and expanded in culture. They are thus an alternative source of somatic cells for reprogramming. UDCs were first reprogrammed using integrating retroviral pMX vectors (Zhou, Benda, Dunzinger, et al., 2012; Zhou, Benda, Duzinger, et al., 2011), and later using non‐integrating episomal plasmids (Steichen et al., 2017; Wang et al., 2017; Xue et al., 2013). However, there is a (residual) risk of integration of episomal vectors into the host genome (Okita et al., 2011; Wang et al., 2017). Plasmid integration can be detected by PCR with specific primers, but integration of fragments can only be excluded by whole genome sequencing. In addition, compared with other reprogramming methods, karyotypic abnormalities may occur more frequently using episomal vectors (Schlaeger et al., 2015).

Sendai virus (SeV) is considered entirely non‐integrating (Nishimura et al., 2011); SeV has also been used successfully for generating hiPSCs from UDCs (Afzal & Strande, 2015; Hildebrand et al., 2016). Since the virus is replication‐deficient, it is normally eliminated by continuous division of the host cells. However, in some cases it has been shown to persist even after multiple passages in culture (Schlaeger et al., 2015); this may adversely affect hiPSC quality and may limit use due to laboratory safety requirements.

Much like SeV, RNA is another “zero footprint” reprogramming vector. Originally messenger (m)RNA was used for reprogramming. Since it is quickly degraded by the intracellular interferon (IFN)α/β‐mediated response to foreign RNA, transfection on 11 consecutive days was required to reprogram UDCs, resulting in high workload and extra costs (Gaignerie et al., 2018). As an alternative, Yoshioka et al. developed a self‐replicative (sr)RNA, which only requires a single transfection for reprogramming skin fibroblasts. The degradation of srRNA is prevented during reprogramming by addition of B18R, which blocks the INF‐y response. Omission of B18R upon emergence of hiPSC‐colonies leads to complete srRNA removal (Yoshioka & Dowdy, 2017; Yoshioka et al., 2013). Recently, an srRNA containing *GFP*, *OCT3/4*, *SOX2*, *KLF4*, and *c‐MYC* was used for reprogramming UDCs (Steinle et al., 2019). However due to an intermediate culture split, reprogramming efficiencies may have been overestimated and hiPSC colonies were possibly of a mixed origin. Moreover, the protocol required B18R protein supplementation for 26 days, making the experiment costly compared to other methods.

Here we describe a method to reprogram UDCs with commercially available srRNA containing the reprogramming factors *OCT3/4*, *SOX2*, *KLF4*, *c‐MYC*, and *GLIS1* (ReproRNA‐OKSGM) (Yoshioka & Dowdy, 2017) with defined media on Matrigel. As the ReproRNA‐OKSGM vector is large (∼16,500 nt), we tested various transfection methods of which nucleofection proved to be the most suitable in terms of required cell number and transfection efficiency. Flow cytometry analysis performed on day 3 allowed quantification of transfection efficiency, enabling termination of an unsuccessful experiment at an early timepoint. B18R protein is added to the cells for 12 days following transfection. Our experiments using UDCs isolated from three adult donors demonstrated that 4‐82 hiPSC colonies (corresponding to 0.008%‐0.17% reprogramming efficiency) can be generated in a single experiment, despite the relatively low percentage of transfected cells. Due to a lack of an intermediate splitting step, hiPSC colonies are likely to be clonal. UDC‐derived hiPSCs are free of the reprogramming vector and display a normal karyotype. They express typical pluripotency markers and have in vitro trilineage differentiation capacity. We also provide supporting protocols for the characterization of pluripotency by FACS and pre‐labeled antibodies for immunofluorescent staining of derivatives of the three germ layers.

## REPROGRAMMING OF URINE‐DERIVED CELLS USING ReproRNA‐OKSGM

Similar to many other primary cell types it is difficult to transfect UDCs with large vectors using regular lipid‐based transfection. Here we describe a step‐wise feeder‐free protocol to reprogram UDCs with ReproRNA‐OKSGM using electroporation as an alternative transfection method, hence combining a non‐integrating reprogramming vector with a cell source that can be harvested through non‐invasive methods. The first section describes the starting material and how to prepare for the electroporation. In the next set of steps the UDCs are harvested and transfected with ReproRNA‐OKSGM and subsequently cultured until hiPSC colony picking. The final section describes how to quantify the transfection efficiency by flow cytometry.

### Materials

UGCs (see Zhou, Benda, Dunziner, et al., 2012)
Renal Epithelial Cell Growth (REGM)‐medium (Lonza, cat. no. CC‐3190)
Transfection (TF) medium (see recipe)
Matrigel, hESC‐qualified (Corning, cat. no. 354277)
DMEM‐F12 (Gibco, cat. no. 10565018)
ReproRNA‐OKSGM (STEMCELL Technologies, cat. no. 05931)
Dulbecco's phosphate‐buffered saline (DPBS, Gibco, cat. no. 14190‐169)
Trypsin‐EDTA, 0.05% (Gibco, cat. no. 25300054)
0.4% Trypan‐Blue (Invitrogen, cat. no. T10282)
Neon Transfection System 10 µl kit (Invitrogen, MPK1096) containing:
  Resuspension buffer R
  Buffer E
REGM‐medium with B18R (see recipe)
ReproTeSR with B18R (see recipe)
TeSR‐E8 (STEMCELL Technologies, cat. no. 05990)
FIX & PERM cell permeabilization kit (Invitrogen, cat. no. GAS003) containing:
  Medium A
Medium BFACS buffer (see recipe)
Anti‐OCT3/4 Isoform A‐PE antibody (Miltenyi Biotec, cat. no. 130‐105‐606, RRID: AB_2653084)

Serological pipettes (5‐, 10 ml, sterile)
Pipette tips (10‐, 200‐, 1,000 µl, sterile, RNase‐/DNase‐free)
Pipettes (0.5 µl to 1,000 µl)
Culture plates (12‐well and 6‐well, clear, sterile)
37°C, 5% CO_2_ humidified incubator
Neon Transfection System (Invitrogen, MPK5000)
Tubes (disposable, 15 ml, sterile)
Centrifuge
Cell counter
Eppendorf tubes (disposable, 1.5 ml, sterile, RNase‐/DNase‐free)
Falcon round‐bottom test tube with cell strainer (Corning, cat. no. 352235)
Flow cytometer

### Treatment of UDCs before transfection

**Figure 1:**
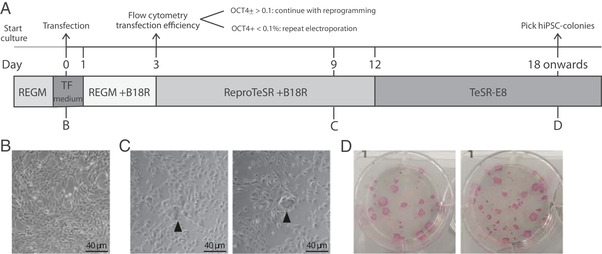
(**A**) Schematic of reprogramming experiment. (**B**) UDCs before transfection. (**C**) Morphology of UDCs at day 9 after transfection. Arrowhead: clusters of UDCs undergoing reprogramming. (**D**) Alkaline phosphatase staining of hiPSC‐colonies at day 21 after transfection (6‐well format).

1 Culture early passage UDCs REGM‐medium in one well of a 6‐well culture plate until 80%‐90% confluent. Before reprogramming make sure that the UDCs are mycoplasma negative by using a standard testing kit.
  Isolation of UDCs according to Zhou, Benda, Dunzinger, et al. (2012).
2 Refresh UDCs with 1.5 ml transfection (TF) medium 1 h prior to harvesting of the UDCs (step 11) (Fig. 1A/B).

### Preparation of Matrigel‐coated wells

3 Thaw a Matrigel aliquot on ice and dilute with cold DMEM‐F12 according to the manufacturer's instructions.
4 Add 1 ml of the diluted Matrigel into one well of 6‐well plate and 0.5 ml into 2 wells of a 12‐well plate, respectively.
5 Incubate for at least 30 min at room temperature (RT).
6 Remove the Matrigel solution and add 1.5 ml TF medium to each coated well of the 6‐well and 0.75 ml to each coated well of the 12‐well‐plate.
7 Place in the incubator until further use.

### Setting up of the NEON transfection system

8 Set up the NEON pipette station according to manufacturer's instructions.
9 Enter the following transfection parameters manually: 1,200 V, 50 ms, 1 pulse.

### Thawing of ReproRNA‐OKSGM

10 Thaw the ReproRNA‐OKSGM on ice.

### Harvesting of UDCs for transfection

11 Wash the UDCs with 2 ml DPBS (RT).
12 Add 0.5 ml of 0.05% Trypsin‐EDTA and place in the incubator for 4 min.
  Cells should be completely detached after 4 min incubation with Trypsin‐EDTA. If still adherent, gently tap the plate to loosen the cells.
13 Add 2 ml REGM medium (RT) to the cell suspension, transfer into a 15‐ml conical tube, and centrifuge at for 3 min at 200 × *g*, RT.
14 Discard the supernatant and gently resuspend the pellet in 2 ml TF medium.
15 Take 10 µl of the cell suspension and mix with 10 µl of 0.4% Trypan Blue.
16 Count the number of live (unstained) UDCs using a cell counter. Refer to the manufacturer's guidelines for instructions.
17 Take an aliquot corresponding to 2.4 × 10^5^ live cells and centrifuge for 3 min at 200 × *g*, RT.

### Transfection of UDCs

18 Carefully remove the supernatant without disturbing the cell pellet.
  It is important to remove as much volume as possible, to minimize the dilution of Resuspension buffer R in the next step.
19 Resuspend UDCs in 22 µl of resuspension buffer R (Neon Transfection kit).
20 Transfer 11 µl of the cell suspension into a 1.5‐ml Eppendorf tube. Keep the remaining cell suspension at RT.
21 Add 1 µl of ReproRNA‐OKSGM directly into the cell suspension and mix well by pipetting gently up and down.
22 Aspirate 10 µl of the cell suspension/ReproRNA mix from step 21 with the NEON‐pipette, avoid air bubbles.
  Any air bubble in the tip causes arcing, which can result in reduced or failed electroporation of the UDCs.
23 Insert the Neon‐pipette vertically in the Neon‐tube containing 3 ml Buffer E (Neon Transfection kit) in the Neon Pipette Station (as prepared in step 8).
24 Electroporate the cells using the parameters of step 9. (Fig. 1A)
25 Remove the Neon‐pipette from the station and transfer the electroporated cells into a 1.5‐ml Eppendorf tube.
26 Plate 5 µl of the transfected UDCs into the Matrigel‐coated 6‐well plate and 5 µl into one well of the 12‐well plate with prewarmed TF medium from step 7. Distribute the cells by gently rocking the plate.
27 Plate 5 µl of untransfected cells from step 19 in the remaining well of the 12‐well plate. Distribute the cells by gently rocking the plate.
28 Incubate the cells at 37°C and 5% CO_2_ without disturbing them for the next 24 hr._._

### Reprogramming of transfected UDCs

29 Refresh the cells 24 and 48 hr post‐transfection with 1.5 ml REGM‐medium with B18R for the 6‐well plate and 0.75 ml REGM‐medium with B18R for the 12‐well plate.
  Attached single cells should be equally distributed throughout the well.
30 Replace REGM‐medium with B18R with 1.5 ml ReproTeSR+B18R at 72 hr post‐transfection for the 6‐well plate. Refresh cells daily until day 11. For the cells in the 12‐well plate, proceed with step 32.
  Small groups of cells undergoing reprogramming that are surrounded by regular UDCs can be observed from day 7 after transfection (see Fig. 1C).
31 From day 12: refresh cells with 2 ml TeSR‐E8 daily until hiPSC colonies are ready for picking.
  At this timepoint wells are often fully confluent with non‐reprogrammed UDCs surrounding newly formed hiPSC colonies. This will not compromise the growth of the hiPSC‐colonies. However, removal of UDCs around hiPSC colonies by gentle scraping with a pipette tip can accelerate outgrowth of the hiPSC‐colony.
  hiPSC colonies are ready for picking and further expansion around day 18‐21 post transfection (Fig. 1D).

### Flow cytometry analysis to quantify UDC transfection with ReproRNA‐OKSGM

32 Wash both wells of the 12‐well plate (transfected and untransfected UDCs) with 1 ml DPBS.
33 Add 0.25 ml of 0.05% Trypsin‐EDTA to each well and incubate for 4 min at 37°C.
  Cells should be completely detached after 4 min incubation with Trypsin‐EDTA. If still adherent, gently tap the plate to loosen the cells.
34 Add 1 ml of REGM‐medium to each well and transfer the cell suspensions into a 15‐ml tube, prelabeled with either + (transfected‐) or – (untransfected).
35 Centrifuge for 3 min at 200 × *g*, RT.
36 Remove the supernatant, resuspend the pellet in 200 µl Medium A (FIX and PERM permeabilization kit) and incubate for 15 min at RT.
37 Add 3 ml FACS buffer and centrifuge the cells for 5 min at 300 × *g*, RT.
38 Remove the supernatant and resuspend the cell pellet in 100 µl Medium B (FIX and PERM permeabilization kit).
39 Add 2 µl of conjugated anti‐OCT3/4 antibody (1:50) and incubate for 20 min at RT in the dark.
40 Add 3 ml of FACS buffer and centrifuge for 5 min at 300 × *g*, RT.
41 Remove the supernatant and wash the cells with 3 ml FACS buffer. Centrifuge for 5 min at 300 × *g*, RT.
42 Resuspend the pellet in 200 µl FACS buffer and filter the cell suspension by using a cell strainer in the lid of a Falcon round‐bottom test tube.
43 Measure the percentage of OCT3/4^+^ cells with a flow cytometer. Use the untransfected cells as a negative control.
  If the percentage of OCT3/4^+^ is below 0.1%, discontinue the reprogramming experiment and repeat the electroporation.

## Support Protocol 1 DETERMINATION OF THE PLURIPOTENCY STATUS OF hiPSCs BY FLOW CYTOMETRY

This method describes a flow cytometry‐based characterization of the pluripotency status of undifferentiated hiPSCs, by measuring the expression of pluripotency markers.

### Additional Materials (also see Basic Protocol)

hiPSC cultures (see the Basic Protocol)
Gentle Cell Dissociation Reagent, GCDR (STEMCELL Technologies, cat. no. 07180)
FIX and PERM cell permeabilization kit (Invitrogen, cat. no. GAS003) containing:
  Medium A
  Medium B
FACS buffer (see recipe)
Anti‐OCT3/4‐BV421 antibody (BD Biosciences, cat. no. 565644, RRID: AB_2739320)
Anti‐Nanog‐PE antibody (BD Biosciences, cat. no. 560483, RRID: AB_1645522)
Anti‐SSEA4‐FITC antibody (Miltenyi, cat. no. 130‐098‐371, RRID: AB_2653517)

### Flow cytometry of pluripotency markers

Culture hiPSCs according to standard procedures in a 6‐well plate. On the day of passaging, use 1 × 6‐well for flow cytometry to measure expression of pluripotency markers. You can take along primary cells (e.g., skin fibroblast, HACAT, but do not use UDCs as they express SSEA4 at high levels) as a negative control and Fluorescence Minus One controls for setting up the flow cytometer.

1 Remove culture medium from the hiPSC cultures and add 1 ml GCDR; incubate for 7 min at 37°C.
2 Pipette vigorously up and down several times with a 1000‐μl pipette to dislodge the cells and generate a single‐cell suspension.
3 Check cell suspension under a brightfield microscope; if cell aggregates persist, repeat step 2.
4 Add 4 ml DMEM/F12 to the cell suspension and transfer into a 15‐ml tube.
5 Take 10 µl of the cell‐suspension and mix with 10 µl of 0.4% Trypan Blue.
6 Count the number of live cells using a cell counter according to the manufacturer's instructions.
7 Take the volume of the cell suspension corresponding to 1 × 10^5^ cells and centrifuge for 3 min at 200 × *g*, RT.
8 Discard the supernatant, resuspend the cells in 200 µl Medium A (FIX and PERM permeabilization kit), and incubate 15 min at RT.
9 Add 3 ml FACS buffer to the cells and centrifuge for 5 min at 300 × *g*, RT.
10 Remove the supernatant and resuspend the cell pellet in 80 µl Medium B (FIX and PERM permeabilization kit).
11 Add 4 µl conjugated anti‐OCT3/4 antibody (1:25), 4 µl conjugated anti‐SSEA4 antibody (1:25), and 20 µl conjugated anti‐Nanog antibody (1:5) and incubate for 60 min at RT in the dark.
12 Add 3 ml FACS buffer to the cells and centrifuge for 5 min at 300 × *g*, RT.
13 Wash the cells with 3 ml FACS buffer and centrifuge for 5 min at 300 × *g*, RT.
14 Resuspend the cells in 200 µl FACS buffer and filter using the cell strainer of a Falcon round‐bottom test tube.
15 Measure the percentage of OCT3/4‐/Nanog‐/SSEA4‐triple positive cells with a flow cytometer.
  Set up the flow cytometer using the appropriate controls. At least 75% of the cells should be positive for all three markers.

## Support Protocol 2 CHARACTERIZATION OF FUNCTIONAL PLURIPOTENCY OF hiPSCs BY IMMUNOFLUORESCENT STAINING WITH PRE‐LABELED ANTIBODIES

The method below describes a way to check the functional pluripotency of hiPSCs by immunofluorescent staining after directed short‐term differentiation into derivatives of endo‐, ecto‐, and mesoderm.

### Additional Materials (also see Basic Protocol)

100% Ethanol
Stemdiff Trilineage Differentiation Kit (STEMCELL Technologies, cat. no. 05230)
2% Paraformaldehyde (PFA; see recipe)
Permeabilization/Blocking solution (see recipe)
4% Normal Swine Serum (4% NSS; see recipe)
Conjugated antibodies (Cell Signaling Technologies, custom‐made, pre‐labeled, see Table 1)
0.05% Tween/PBS (see recipe)
DAPI (Invitrogen, cat. no. D3571)
MilliQ water
ProLong Gold Antifade Mountant (Invitrogen, cat. no. P36930)

Glass coverslips (13‐mm diameter)
Tweezers
Bunsen burner
Culture plates (24‐well, sterile, clear)
Glass microscope slides
(Confocal) Fluorescent microscope

**Table 1.** Pre‐Conjugated Antibody‐List Used for Support Protocol 2

Antibody	Cat. no.	Source	Isotype	Conjugated with	Germ layer
anti‐FAPB7	D8N3N	Rabbit	IgG	Alexa555	Ectoderm
anti‐PAX6	D3A9V	Rabbit	IgG	Alexa647	Ectoderm
anti‐Nestin	10C2	Mouse	IgG1	Alexa488	Ectoderm
anti‐FOXA2	D56D6	Rabbit	IgG	Alexa555	Endoderm
anti‐EOMES	D8D1R	Rabbit	IgG	Alexa488	Endoderm
anti‐GATA4	D3A3M	Rabbit	IgG	Alexa647	Endoderm
anti‐Vimentin	D21H3	Rabbit	IgG	Alexa647	Mesoderm
anti‐CDX2	D11D10	Rabbit	IgG	Alexa555	Mesoderm
anti‐Brachyury	D2Z3J	Rabbit	IgG	Alexa488	Mesoderm

### Matrigel coating of coverslips

1 Sterilize a coverslip by dipping it into 100% ethanol using tweezers and subsequent flaming.
2 Place the sterile coverslip in a well of a 24‐well plate. Each germ layer differentiation requires one well with a coverslip.
  Use the same plate for meso‐ and endoderm differentiation (5 days) and a separate plate for ectoderm differentiation (7 days).
3 Thaw a Matrigel aliquot on ice and dilute with cold DMEM‐F12 according to the manufacturer's instructions.
4 Add 330 µl of the diluted Matrigel onto each coverslip.
  Make sure that the coverslip is completely covered with Matrigel. Sometimes coverslips need to be pushed down using a pipette tip.
5 Incubate the plates for at least 30 min at RT before use.

### Trilineage differentiation and fixation of coverslips

6 Plate undifferentiated hiPSCs on Matrigel‐coated coverslips and perform Trilineage differentiation according to the manufacturer's instructions.
7 At the end of differentiation (day 5 for meso‐ and endoderm and day 7 for ectoderm), remove medium from the coverslips and gently wash cells with 1 ml DPBS.
8 Remove DPBS and add 1 ml of 2% PFA to the coverslips; incubate for 30 min at RT.
9 Remove 2% PFA and gently wash cells once with 1 ml DPBS.
10 Add 1 ml DPBS to the coverslips and proceed with the immunofluorescence staining.
  If necessary, fixed cells can be stored for several weeks at 4°C before proceeding with the immunofluorescent staining.

### Immunofluorescent staining of trilineage differentiation

11 Wash the coverslips once with 200 µl DPBS.
12 Remove DPBS and add 80 µl Permeabilization/Blocking solution to each coverslip and incubate 60 min at RT.
13 Prepare antibody mix for all three germ layers by diluting the antibodies in 4% NSS according to Table 2.
14 Wash the coverslips once with 200 µl DPBS.
15 Add 80 µl of the corresponding antibody‐mix to the coverslips (Table 1/2) and incubate for 60 min, at RT, in the dark.

**Table 2.** Dilution Factors Antibodies for Support Protocol 2

Antibody mix	Components	Dilution	Volume (µl)
Ectoderm	anti‐FAPB7	1:100	1
	anti‐PAX6	1:200	0.5
	anti‐Nestin	1:200	0.5
	4% NSS		98
Endoderm	anti‐FOXA2	1:500	0.2
	anti‐EOMES	1:100	1
	anti‐GATA4	1:200	0.5
	4% NSS		98.3
Mesoderm	anti‐Vimentin	1:400	0.25
	anti‐CDX2	1:500	0.2
	anti‐Brachyury	1:200	0.5
	4% NSS		99

16 Incubate the coverslips three times, each time with 200 µl of 0.05% Tween/PBS for 10 min in the dark.
17 Dilute DAPI stock‐solution (1 mg/ml) 1:500 in DPBS.
18 Add 80 µl of the diluted DAPI to the coverslips and incubate for 5 min, at RT, in the dark.
19 Wash the coverslips once with 200 µl MilliQ water.
20 Put a droplet of ProLong Gold (∼10 µl) on a pre‐labeled glass microscope slide.
21 Remove the MilliQ water from the coverslips and mount coverslip upside down onto the microscope slide.
22 Dry the microscope slide for at least 24 hr in the dark.
23 Image the slides with a (confocal) fluorescent microscope.

## REAGENTS AND SOLUTIONS

### FACS buffer

Dissolve bovine serum albumin (BSA; Sigma, cat. no. A8022) in DPBS (Gibco, cat. no. 14190‐169) at 5 mg/ml. Add EDTA (0.5 M EDTA; ThermoFisher, cat. no. AM9260G) to a final concentration of 0.2 mM. Store FACS buffer up to 4 weeks at 4°C.

### Normal swine serum (NSS), 4%

Dilute NSS (Jackson ImmunoResearch Laboratories, cat. no. 014‐000‐121) at 1:25 in DPBS (Gibco, cat. no. 14190‐169)
Prepare fresh

### Paraformaldehyde (PFA), 2%

*8% PFA (2 L)*
Heat 1,500 ml MilliQ water to 80°C
Weigh 160 g Paraformaldehyde (Merck, cat. no. 1.04005.1000) in an Erlenmeyer flask
Place the Erlenmeyer flask on a magnetic stirrer in a chemical hood
Add 1,500 ml MilliQ water of ∼74°C to the Paraformaldehyde and stir 5 min until dissolved
Adjust the pH to 7.4 using 5 M NaOH (Merck, cat. no. 1.06498.1000)
Let the solution cool down while stirring
Add 500 ml MilliQ
Sterilize by using a 0.22‐µm filter
Store up to 3 months at 4°C
*2% PFA*
Dilute 8% PFA 1:4 with the phosphate buffer
Prepare fresh

### Permeabilization/blocking solution

Prepare a 0.1% Triton X‐100/DPBS solution by diluting Triton X‐100 (Sigma, cat. no. T8787) at 1:1,000 with DPBS (Gibco, cat. no. 14190‐169)
Dilute NSS (Jackson ImmunoResearch Laboratories, cat. no. 014‐000‐121) at 1:25 with the 0.1% Triton X‐100/DPBS solution
Prepare fresh

### Phosphate buffer

Prepare a 0.2 M solution of NaH_2_PO_4_⋅H_2_O (Merck, cat. no. 1.06346.1000) in MilliQ water
Prepare a 0.2 M solution of Na_2_HPO_4_⋅2H_2_O (Gerbu, cat. no. 1309‐1000) in MilliQ water
Add NaH_2_PO_4_⋅H_2_O (acid) solution to the Na_2_HPO_4_⋅2H_2_O (base) solution until it reaches a pH of 7.4
Store up to 3 months at 4°C

### REGM‐medium with B18R

Prepare the REGM‐medium according to manufacturer's instructions (REGM Bulletkit, Lonza, cat. no. 3190)
Thaw B18R protein at RT and add at 1:2,500 to the medium
  Prepare aliquots of the B18R protein if you do not use the complete volume. Store aliquots at −80°C. Avoid repeated freeze‐thaw cyclesm
Warm medium to RT before use.
  Complete REGM‐medium with B18R can be stored up to 1 week at 4°C.

### ReproTeSR with B18R

Prepare ReproTeSR according to manufacturer's instructions (STEMCELL Technologies, cat. no. 05926)
Thaw B18R protein at RT and add at 1:2,500 to the medium
  Prepare aliquots of the B18R protein if you do not use the complete volume. Store aliquots at −80°C. Avoid repeated freeze‐thaw cycles.
Warm medium to RT before use.
  Complete ReproTeSR with B18R can be stored up to 1 week at 4°C.

### Transfection (TF) medium

Thaw REGM Singlequots, except GA‐1000 (antibiotic) (REGM Singlequots kit, no GA‐1000. Lonza, cat. no. CC4127) on ice
Add REGM Singlequots, except GA‐1000, to the Renal Epithelial Cell Growth Basal Medium (REBM. Lonza, Cat.no CC3191)
  IMPORTANT: The TF medium must not contain any antibiotics as this can lead to increased cell‐death. (Prepare aliquots of the Singlequots when you are not using the complete volume. Store aliquots at −20°C. Avoid repeated freeze‐thaw cycles.)
Thaw B18R protein at RT and add at 1:2,500 to the medium
  Prepare aliquots of the B18R protein if you do not use the complete volume. Store aliquots at −80°C. Avoid repeated freeze‐thaw cycles.
Warm medium to RT before use.
  Complete TF medium can be stored up to 1 week at 4°C.

### Tween/PBS, 0.05%

Pipette 49.975 ml of DPBS (Gibco, cat. no. 14190‐169) into a 50‐ml tube
Cut the end of a 100‐μl pipette tip to enlarge the opening
Pipette up 25 µl of Tween‐20 (Merck, cat. no. 8.22184.0500) with the pre‐cut pipette tip
  Tween‐20 is very viscous, pipette slowly to ensure aspirating the complete amount.
Add the Tween‐20 to the tube containing the DPBS (drop the used pipette tip inside of the tube)
Put the 50‐ml tube on a tube rotator until the Tween‐20 is properly dissolved
Store for several months at RT

## COMMENTARY

### Background Information

Zhou et al. were the first to show that cells extracted and expanded from urine samples can be used for reprogramming (Zhou, Benda, Dunzinger, et al., 2012; Zhou, Benda, Duzinger, et al., 2011). These so‐called urine‐derived cells (UDCs) are a heterogeneous population, which originate mainly from the renal epithelium. Their identity is based on high expression levels of several epithelial markers (e.g., Occludin and Claudin1) and renal tubular markers (e.g., CD13 and NR3C2) (Dorrenhaus et al., 2000; Rahmoune et al., 2005; Zhou, Benda, Duzinger, et al., 2011). However, expression of urothelial markers and stem cell markers have also been described (Bharadwaj et al., 2011; Zhang et al., 2008).

Zhou et al. were the first to reprogram UDCs with retroviral pMX vectors with an efficiency of 0.1%‐4%. The use of retroviruses for reprogramming is unfavorable because stable integration of the retroviral DNA can lead to incomplete transgene silencing or re‐activation under certain conditions (Koyanagi‐Aoi et al., 2013; Okita, Ichisaka, & Yamanaka, 2007). The latter has been shown to negatively affect the differentiation capacity of hiPSCs and can even cause malignancy (Bouma et al., 2017). Moreover, hiPSCs generated with retroviral vectors have high aneuploidy rates (Schlaeger et al., 2015).

Since the first description of UDCs as a cell source for reprogramming, multiple efforts have been made to reprogram UDCs using different reprogramming methods. Several groups generated hiPSCs from UDCs with episomal plasmids (Steichen et al., 2017; Wang et al., 2017; Xue et al., 2013); however, episomal DNA can occasionally integrate in the host‐genome and may increase aneuploidies (Okita et al., 2011; Schlaeger et al., 2015; Wang et al., 2017). As a truly non‐integrative approach, Sendai virus has been used for reprogramming of UDCs (Afzal & Strande, 2015; Hildebrand et al., 2016). However, persistence of Sendai virus vectors in hiPSCs of relatively high passage has been observed (Afzal & Strande, 2015; Schlaeger et al., 2015). Commercially available SeV contains temperature‐sensitive mutations in a subset of the vectors requiring incubation of hiPSCs at 38°‐39°C for 5 days for clearance. However, hiPSCs might be sensitive to culture at elevated temperatures. As an alternative non‐integrating vector mRNA has been successfully deployed for the reprogramming of somatic cells including UDCs (Gaignerie et al., 2018; Warren et al., 2010). Due to the low stability of exogenous mRNA the reprogramming procedure requires transfections on multiple consecutive days and is, therefore, laborious, error‐prone and expensive.

To overcome these hurdles, Yoshioka et al. (2013) developed a self‐replicative (sr)RNA. The original srRNA version is based on a single, synthetic Venezuelan Equine Encephalitis (VEE) RNA replicon encoding the reprogramming factors *OCT3/4*, *KLF4*, *SOX2*, and *c‐MYC* or *Glis1*. The continuous expression of the reprogramming factors is ensured by self‐replication of the vector and the suppression of RNA degradation by B18R supplementation (Alcami, Symons, & Smith, 2000; Colamonici, Domanski, Sweitzer, Larner, & Buller, 1995). A single transfection with the srRNA is sufficient to successfully reprogram fibroblasts into hiPSCs (Yoshioka et al., 2013). An improved version of the srRNA vector contains all five factors (Yoshioka & Dowdy, 2017).

In 2019, hiPSCs were generated from UDCs using an srRNA with the four Yamanaka factors as well as GFP to monitor transfection efficiency (Steinle et al., 2019). Between 3‐25 hiPSC colonies were obtained after a single transfection with lipofectamine. However, the protocol included an intermediate splitting step after transfection, possibly leading to an overestimation of transfection efficiency and the emergence of non‐clonal hiPSC colonies.

Our protocol is based on a commercially available and improved srRNA version containing the reprogramming factors *OCT3/4*, *KLF4*, *SOX2*, *GLIS1* and *c‐MYC*. Only 1.2 × 10^5^ cells are required for the actual electroporation and the total number of 2.4 × 10^5^ cells for the whole experiment is easily obtained by culturing UDCs in a single well of a 6‐well plate. Fewer population doublings reduce the risk of cells becoming senescent, which is known to be counteractive for successful reprogramming in general and has previously been seen for UDCs. (Li et al., 2016). Moreover, in our protocol B18R protein supplementation is only required for 12 days, which significantly reduces the costs compared to other protocols. In addition, we found that puromycin selection as described for fibroblasts was not necessary for UDC reprogramming resulting in a simplified experimental procedure. By measuring the percentage of OCT3/4^+^ cells 72 hr post‐transfection our protocol provides an early checkpoint in order to determine whether an experiment is likely to be successful. Finally, we provide straightforward protocols for the basic characterization of the pluripotency status and differentiation capacity of hiPSCs.

### Critical Parameters

UDCs need to be at an early passage and highly proliferative to enable proper reprogramming. Otherwise, the reprogramming efficiency decreases dramatically (Li et al., 2016). It is therefore important to start the UDC isolation with sufficiently large volumes of urine (>100 ml) and to process the urine immediately after collection. On average we obtain ∼5 UDC colonies per 100 ml of urine; however, there is variability between donors and even separate samples from the same donor can give different isolation efficiencies. When culturing UDCs, they should be passaged at a ratio 1:4 when reaching 80%‐90% confluency to ensure proper growth. Normally, it takes 3‐5 days before they reach the required confluency again.

UDCs should be transfected as a single‐cell suspension to ensure proper transfection using the Neon‐system. After centrifugation the culture medium should be removed carefully, without disturbing the cell pellet. We usually remove the supernatant just above the pellet with a pipette tip instead of aspiration by vacuum. Moreover, it is important to avoid any arcing using the Neon system. When a spark has been observed during transfection, transfection efficiency might be reduced. We therefore recommend to always check the transfection efficiency by flow cytometry after 72 hr.

We found that despite low transfection efficiencies a minimum of four hiPSC colonies/clones were obtained per reprogramming experiment. In general, we consider three hiPSC clones per donor as sufficient. At transfection efficiencies of <0.1% we were never able to obtain any hiPSC colonies. In this case we recommend aborting the ongoing experiment and repeating the electroporation in order to save both time and money.

At day 12 during UDC reprogramming we switch culture medium from ReproTeSR + B18R to TeSR‐E8 medium, to promote outgrowth of the formed hiPSC‐colonies. Although small hiPSC‐colonies might already be observed at an earlier timepoint, it is important not to start with TESR‐E8 before day 12 as hiPSC lines established under those conditions seem to have a bias for spontaneous neuroectodermal differentiation.

### Troubleshooting

Problems that may arise at different steps and their possible solutions are listed in Table 3.

**Table 3.** Troubleshooting Guide

Step	Problem	Solution
Basic Protocol, step 29	Increased cell‐death after transfection	Some degree of cell‐death is expected after the transfection of the cells using these electroporation parameters. However, there should be attached cells 24 hr post plating.
Basic Protocol, step 31 (annotation)	No hiPSC colonies appearing (with transfection efficiency >0.1%)	Ensure your UDCs are low passage and proliferative.
Basic Protocol, step 31 (annotation)	hiPSC colonies remain small due to fully confluent non‐reprogrammed UDCs	Carefully remove non‐reprogrammed UDCs surrounding the hiPSC colony by scraping with a pipette‐tip without damaging the colony. Change medium and keep in culture for a couple of days until hiPSC colony is ready for picking.
Basic Protocol, step 43	Transfection efficiency below 0.1%	Repeat the experiment ensuring that: TF medium does not contain antibiotics. Cells appeared as single cells before transfection Transfect the correct number of viable cells. The srRNA has not been degraded. Use RNase‐free tubes and tips. Thaw srRNA on ice shortly before use. Avoid repeated freezing and thawing. No error or spark was observed using the Neon transfection system
Support Protocol 1, step 15	Low fluorescent intensity for one or more antibodies	There might be batch‐to‐batch variation for pre‐labeled antibodies. Test different batches and determine the optimal dilution.
Support Protocol 2, step 7 & 8	Cells are detached from coverslips after this step	Avoid adding DPBS and 2% PFA at high speed directly onto the cells. As the cells differentiate and become dense, they tend to detach more easily (as a sheet). Add fluids gently against the well wall.

### Understanding Results

#### Reprogramming time‐course for UDCs

Small clusters of cells undergoing reprogramming will be visible from day 7 onwards. Compared to the original UDCs these show morphological changes, such as formation of compact cell clusters, high nucleus‐to‐cytoplasm ratios and few clearly visible nucleoli. No clear borders are observed yet for all clusters (Fig. 1A−C). When medium is switched to TeSR‐E8 at day 12, some clusters will disappear in the following 2‐3 days, whereas others transform into compact hiPSC colonies with defined borders. Outgrowth of hiPSC colonies can be accelerated by manual removal of surrounding non‐reprogrammed UDCs. Usually at day 18‐21 post‐transfection, hiPSC colonies are sufficiently large for manual picking (Fig. 1A/D).

#### Measuring transfection efficiency

**Figure 2:**
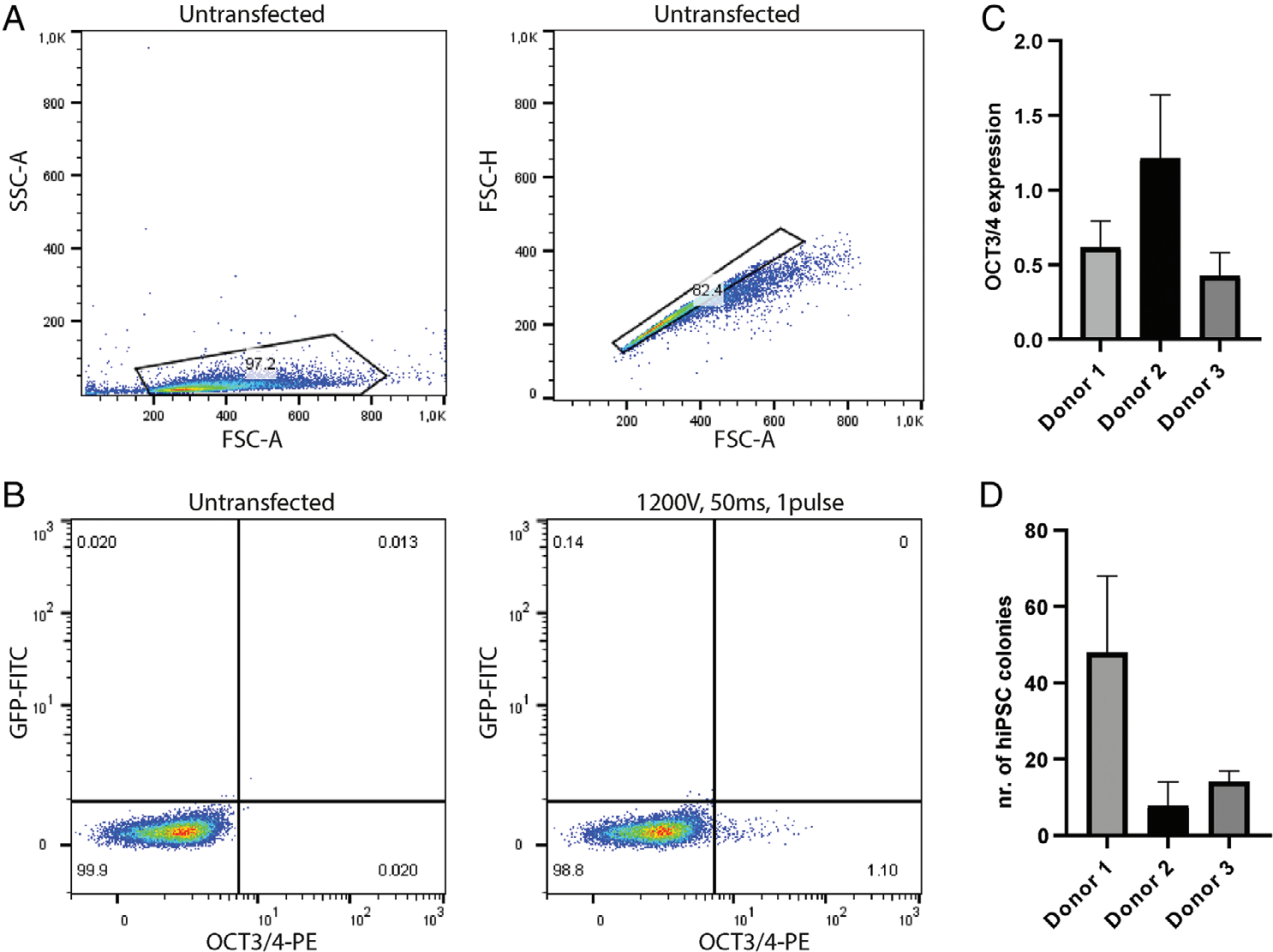
ReproRNA‐OKSGM reprogramming of UDCs. (**A**) Flow cytometry gating strategy for live and single cells, respectively to check transfection efficiency. (**B**) Example of transfection efficiency measured by flow cytometry for donor 1. (**C**) OCT3/4 expression measured by flow cytometry for three different donors, 72 hr after transfection (±SD). (**D**) Number of hiPSC colonies at 21 days after transfection for three different donors (±SD) (Donor 1: *n* = 7, donor 2: *n* = 3, donor 3: *n* = 2).

UDCs seeded in the 12‐well plate after transfection are subjected to flow cytometry to quantify transfection efficiency. This is performed at 72 hr post‐transfection, to enable the cells to recover from the electroporation procedure. Due to the transfection stress many cells will die, resulting in a low number of attached cells the day after transfection. Untransfected cells (negative control) will be confluent after 72 hr in most cases. Percentages of OCT3/4‐expressing cells was in the range ∼0.2%–1.3% (0.64 ± 0.40%), resulting in 4‐86 hiPSC (32 ± 23) colonies for three donors (Fig. 2). Of note, the transfection efficiency is not directly correlated with the reprogramming efficiency but rather seems to be donor‐dependent. However, out of 15 performed reprogramming attempts, three failed due to transfection efficiencies below 0.1% (ranging from ∼0.01% to 0.06%).

#### Measuring the pluripotency status of hiPSCs

**Figure 3:**
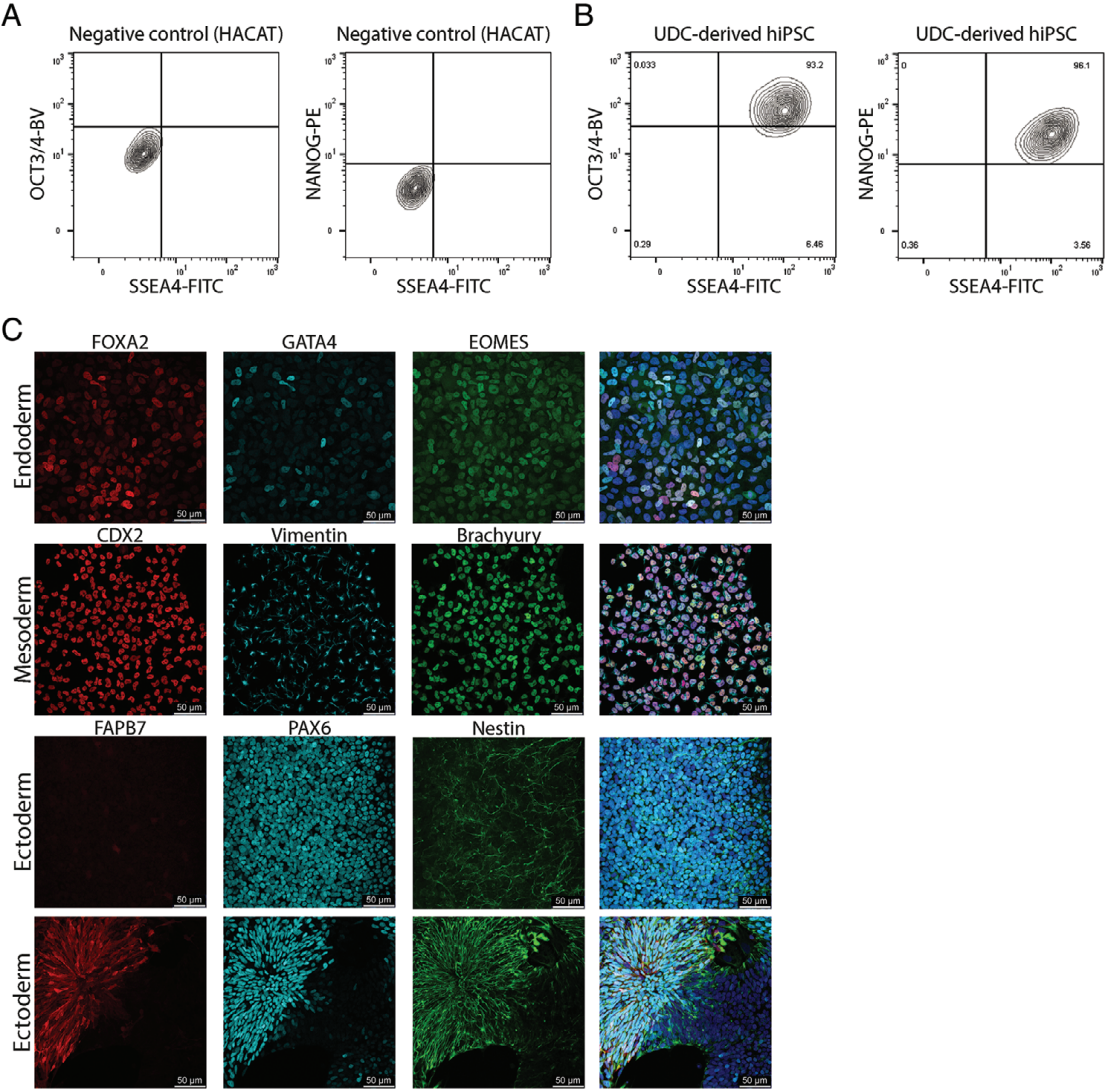
Basic characterization of hiPSC lines. (**A**) Use of an immortalized keratinocyte cell‐line (HACAT) as negative control for setting the gates for measuring pluripotency markers. (**B**) Flow cytometry analysis of pluripotency marker expression in UDC‐derived hiPSCs. (**C**) Examples of immunofluorescence staining after trilineage differentiation of hiPSC‐lines derived from UDCs.

The pluripotency status of undifferentiated hiPSCs can be determined by flow cytometry, measuring the percentage of cells expressing pluripotency markers OCT3/4, Nanog, and SSEA4. In a maintenance culture with little spontaneous differentiation, the majority of cells are OCT3/4‐/Nanog‐/SSEA4 triple‐positive; in general, 75% is regarded as a threshold for high‐quality cells. When cells differentiate they usually first lose the expression of Nanog and OCT3/4, while SSEA4 can remain present for a longer period (Fig. 3A/B).

#### Characterization of functional pluripotency of hiPSCs

For ecto‐ and endoderm differentiation, hiPSCs are plated at a density to reach 80%‐90% confluency on the next day, while the mesoderm coverslips will be 20%‐30% confluent. However, after switching the maintenance medium to differentiation medium, the cells seeded on coverslips for endoderm differentiation may show detaching cells. This only happens on day 1 of the protocol and will not hamper differentiation of the remaining cells. When analyzing the immunofluorescence staining of ectodermal, mesodermal, and endodermal cells we usually obtain the following results: Mesodermal differentiation is homogeneous and most of the cells express Vimentin (cytoplasmic), Brachyury T, and CDX2 (both nuclear). Endoderm differentiation is more heterogeneous with cells expressing a combination of FOXA2, EOMES, and/or GATA4 (all nuclear). The number of positive cells is sometimes lower in comparison with mesodermal differentiation. Ectodermal differentiation is often a mix of 3D structures surrounded by monolayers. Patches of cells expressing PAX6 (nuclear) and Nestin (cytoplasmic) are commonly found whereas expression of FAPB7 (nuclear and cytoplasmic) is less common. (Fig. 3C)

### Time Considerations

#### Basic Protocol

Steps 1‐10: 15 min

Steps 11‐17: 20 min

Steps 18‐28: 5 min

Step 29‐31: ∼21 days

Step 32‐43: 1.5 hr

#### Support Protocol 1

2.5 hr

#### Support Protocol 2

Steps 1‐5: 5 min

Steps 6‐10: 7 days

Steps 11‐23: 2 days
